# Specific Collagen Peptides Improve Bone Mineral Density and Bone Markers in Postmenopausal Women—A Randomized Controlled Study

**DOI:** 10.3390/nu10010097

**Published:** 2018-01-16

**Authors:** Daniel König, Steffen Oesser, Stephan Scharla, Denise Zdzieblik, Albert Gollhofer

**Affiliations:** 1 Department for Nutrition, Institute for Sports and Sports Science, University of Freiburg, Schwarzwaldstr. 175, 79117 Freiburg, Germany; Denise.Zdzieblik@sport.uni-freiburg.de (D.Z.); AG@sport.uni-freiburg.de (A.G.); 2 CRI, Collagen Research Institute GmbH, Schauenburgerstr. 116, 24118 Kiel, Germany; steffen.oesser@cri-mail.org; 3 Independent Reasercher, Salinenstr. 8, 83435 Bad Reichenhall, Germany; SScharla@gmx.de

**Keywords:** osteoporosis, collagen hydrolysate, SCP, bone marker, protein supplementation

## Abstract

**Introduction:** Investigations in rodents as well as in vitro experiments have suggested an anabolic influence of specific collagen peptides (SCP) on bone formation and bone mineral density (BMD). The goal of the study was to investigate the effect of 12-month daily oral administration of 5 g SCP vs. placebo (CG: control group) on BMD in postmenopausal women with primary, age-related reduction in BMD. **Methods:** 131 women were enrolled in this randomized, placebo-controlled double-blinded investigation. The primary endpoint was the change in BMD of the femoral neck and the spine after 12 months. In addition, plasma levels of bone markers—amino-terminal propeptide of type I collagen (P1NP) and C-telopeptide of type I collagen (CTX 1)—were analysed. **Results:** A total of 102 women completed the study, but all subjects were included in the intention-to-treat (ITT) analysis (age 64.3 ± 7.2 years; Body Mass Index, BMI 23.6 ± 3.6 kg/m^2^; T-score spine −2.4 ± 0.6; T-score femoral neck −1.4 ± 0.5). In the SCP group (*n* = 66), BMD of the spine and of the femoral neck increased significantly compared to the control group (*n* = 65) (T-score spine: SCP +0.1 ± 0.26; CG −0.03 ± 0.18; ANCOVA *p* = 0.030; T-score femoral neck: SCP +0.09 ± 0.24; CG −0.01 ± 0.19; ANCOVA *p* = 0.003). P1NP increased significantly in the SCP group (*p* = 0.007), whereas CTX 1 increased significantly in the control group (*p* = 0.011). **Conclusions:** These data demonstrate that the intake of SCP increased BMD in postmenopausal women with primary, age-related reduction of BMD. In addition, SCP supplementation was associated with a favorable shift in bone markers, indicating increased bone formation and reduced bone degradation.

## 1. Introduction

The etiology of osteoporosis includes a lack of physical activity, malnutrition, underlying diseases, drug ingestion and non-modifiable factors, such as ageing, gender, and familiar predisposition. Adequate prevention or therapy of osteoporosis is a very important goal for individual and public health, because osteoporotic bone fractures are responsible for chronic pain, inactivity and invalidity in the elderly. It is estimated that, worldwide, every third women, and one in five men over the age of 50, will sustain an osteoporotic-induced bone fracture [1]. At present, there are a number of therapeutic approaches for the prevention and treatment of osteoporosis. Non-pharmacological approaches, such as daily physical activity, smoking cessation and reduction of alcohol consumption, are very important cofactors in maintaining bone health. In addition, supplementation with calcium and vitamin D is recommended in osteoporosis management, but has not been shown to significantly reduce bone fracture risk. Pharmacological treatment includes substances such as bisphosphonates, human monoclonal antibody therapy and selective estrogen receptor modulators. Bisphosphonates are the most widely-used medication and are designated as the “gold standard” anti-catabolic therapy in fracture prophylaxis. However, although the risk-benefit ratio favors treatment with bisphosphonates, some side effects, such as bisphosphonate-related osteonecrosis of the jaw (BRONJ), gastrointestinal disorders, ulcer of the mucosa, hypocalcaemia or predisposed renal failure have been described in the literature [2,3,4]. Other therapy forms focus on recombinant synthesized hormones or the manipulation of hormone receptors; however, these therapies, may also induce a number of side effects [5,6,7]. Compared to other chronic medications, compliance problems are relatively high in patients taking anti-osteoporotic medication. Although a relationship with acute or chronic side effects has not been clearly established, about 40% of patients with oral bisphosphonates discontinue their medication during the first year of therapy and approximately 75% of patients do so by 5 years [8].

Once osteopenia or osteoporosis has been diagnosed, basic therapies, with increased physical activity, a balanced, calcium-rich diet and the reduction of alcohol and nicotine, may protect against a further loss of bone mineral density (BMD). Nevertheless, these approaches are not likely to induce an improvement in BMD. Hence, effective and compliant therapeutic approaches currently rank very high, particularly in a period of worldwide demographic change.

Preclinical in vitro studies or investigations with rodents have shown that administration of collagen peptides increased the organic component of bones [9], improved bone metabolism as well as bone microarchitecture [10,11,12] and enhanced the biomechanical resistance of vertebrae [13]. Moreover, supplementation with collagen peptides in combination with calcitonin has shown positive effects in postmenopausal women [14]. In the latter study, supplementation with collagen peptides led to a statistically significant decreased excretion of bone collagen breakdown products, in comparison to placebo treatment. Moreover, the effect of therapy with collagen peptides was persistent over a period of at least three months after the last administration, suggesting an anabolic effect of collagen peptide treatment.

Therefore, in the present study, the longer-term effects of a specific bioactive collagen peptide supplementation on BMD of the lower spine and the femoral neck, determined by DXA (dual energy X-ray absorptiometry), were tested. For this purpose, postmenopausal women, aged 46–80 years (mean age 63 years) received 5 g specific collagen peptide (SCP)/day or 5 g maltodextrin as a placebo (control group: CG), in a randomized, placebo-controlled design, for 12 months.

## 2. Subjects and Methods

### 2.1. Subjects and Consort Flow Diagram

**Figure 1:**
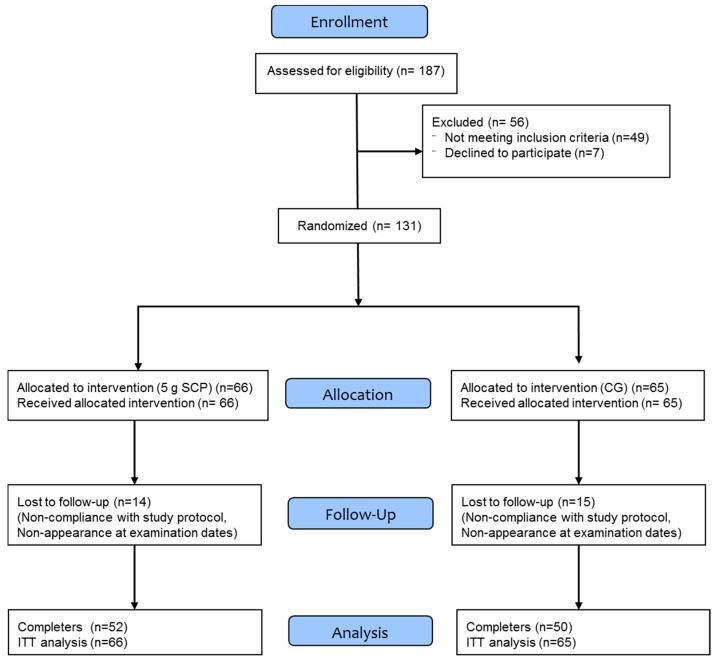
Flow diagram of patient recruitment, randomization and follow up.

A total of 131 postmenopausal women with a reduced bone mineral density (DXA T-score of −1 or lower on either the femoral neck or the lumbar spine) were included in this study. Specific data regarding patient recruitment, allocation and follow-up are presented in Figure 1.

### 2.2. Inclusion Criteria

The inclusion criteria were as follows: female subjects with reduced BMD of the lower spine or the femoral neck, determined by DXA; menopause (amenorrhea for at least 1 year); no diagnosis of any severe chronical disease or co-morbidity; steady state body weight and nutrition; no contraindications for nutraceuticals or protein-rich supplements.

### 2.3. Exclusion Criteria

The exclusion criteria were as follows: medical treatment for osteoporosis within the last year; osteoporosis with high risk for bone fractures and indication for medical treatment; allergy to collagen; medical or endocrinological induced osteoporosis; malignant diseases within the previous 5 years; diabetes mellitus type I or II; renal or liver diseases induced by a high protein load; recent immobilization for several weeks.

### 2.4. Study Design

The investigation was designed as a single-center, prospective, randomized, double-blind, placebo-controlled study. The clinical trial was a phase III study and was carried out according to GCP (good clinical practice) requirements. The study protocol was approved by the ethical committee of the University of Freiburg. All subjects gave written informed consent. The trial was registered with DRKS-ID: DRKS0009708.

The participants of the study were randomly assigned to the treatment group (supplementation with collagen peptides, SCP) or to the placebo group (maltodextrin). Randomization was performed using a random number generator [15]. Blinding of investigators and participants was not lifted until all data were entered, the dataset was secured, and the statistical analyses were performed. The participants had to dissolve the content of one sachet of the investigational product (SCP or maltodextrin) and drink it in a glass of water, before breakfast. Subjects in both groups were instructed by a physician on the beneficial effects of regular physical activity and balanced nutrition on osteopenia or osteoporosis [16,17]. In addition, subjects were encouraged to take calcium and vitamin D supplements in a daily dose of approximately 0.5–0.8 g (depending on weight) and 400–800 IU, respectively. However, the supplements were not prescribed, and the intake was not controlled. Compliance, regarding the intake of the investigational product, was checked by the collection of unused supplements. In addition, subjects were asked to keep daily records about side effects or other problems related to the supplements. In addition, blood samples were taken at the beginning and the end of the study for analysis of bone markers and to evaluate the safety of the product and to verify adverse reactions.

**Figure 2:**
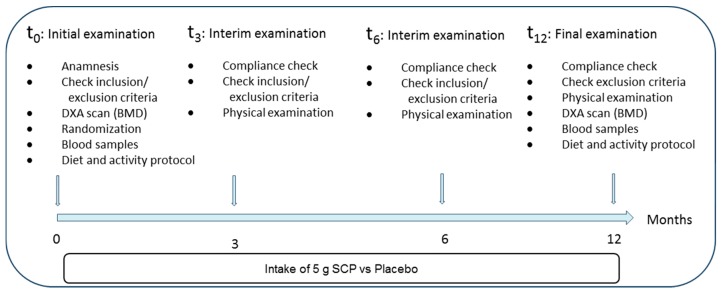
Procedure of the clinical trial and points of examinations. BMD, bone mineral density; DXA, dual energy X-ray absorptiometry.

The study was conducted over a total timeframe of 12 months. All phases of the study are summarized in Figure 2.

### 2.5. DXA

BMD of the lower lumbar spine (L1–L4) and the femoral neck was measured before and after the 12-month study period using DXA (Stratos DR 2D Fan Beam, Degen Medizintechnik, Heppenheim, Germany).

### 2.6. Dietary Behavior and Physical Activity

Dietary behavior, with special reference to macronutrients (fat, carbohydrate, protein), calcium and vitamin D, was evaluated before and at the end of the study, by using a 4-day nutritional protocol. Subjects were asked to fill out the protocol using household measurements on 3 working days and one day off work. The protocols were analyzed using PRODI 6.0 (Prodi, Stuttgart, Germany). Physical activity was assessed with an evaluated questionnaire, in German language, in which the amount and intensity of physical activity was queried, and the total amount of calories burned by these activities was calculated [18].

### 2.7. Protein Supplementation

For this study, a mixture of specific bioactive collagen peptides (SCP) with a mean molecular weight of approx. 5 kDa, derived from a complex multi-step hydrolysis of collagen, was used (FORTIBONE^®^, GELITA AG, Eberbach, Germany). The sachets containing 5 g SCP or placebo (maltodextrin, CARGILL, Paris, France) were identical in appearance and the products were equal in flavor and texture.

### 2.8. Sample Size

The sample size for the study was calculated on the basis of the recent statistical publication from the Center of Disease Control (CDC) of March 2012 [19]. A power analysis was performed, based on the assumption that the bone mineral density of postmenopausal women between 50–59 years was 0.99 ± 0.1 g/cm^2^, as described by CDC. Considering the fact that a therapy with bisphosphonates increased bone density by about 5%, similar to findings by Adam et al. [14], for SCP a hypothetical growth to 1.04 g/cm^2^ was expected. With the standard deviation of 0.1 g/cm^2^ and an intended test power of 80% with a significance level of α = 0.05 and a calculated drop-out rate of 10%, the number of 64 participants per study group was calculated and considered sufficient.

### 2.9. Endpoints

The primary endpoint of this study was defined as comparing differences in bone mineral density (BMD) of the spine (L1–L4) between both study groups (SCP versus placebo). The second primary outcome was defined as changes in BMD of the femoral neck. Changes in bone metabolism were evaluated using the bone biomarkers, amino-terminal propeptide of type I collagen (P1NP) and C-telopeptide of type I collagen (CTX 1). Amino-terminal propeptide of type I collagen (P1NP) was assessed as an indicator of bone formation, whereas C-telopeptide of type I collagen (CTX 1) was measured as marker for bone resorption. Bone turnover was calculated by comparing the number of biomarkers (in ng/mL) in the plasma samples at the end of the study (t_12_), to the value recorded at the beginning of the study (t_0_). An in vitro enzyme immunoassay was used for the quantitative analyses of CTX 1 (BlueGene Biotech., Shanghai, China) and P1NP (Cloud-Clone Corp., Houston, TX, USA). The ELISA tests were performed according to the respective instruction manuals. The sensitivities of the tests were indicated as 12.5 ng/mL and 0.91 ng/mL, respectively.

### 2.10. Statistical Methods

All quantitative parameters are presented as mean ± SD. Statistical analyses were performed using the Statistical Package for the Social Sciences Software (IBM SPSS Statistics 23, IBM, Armonk, NY, USA).

The whole statistical evaluation was based on the intention-to-treat population (ITT). Missing values of all test parameters after the 12-month intervention were completed by a linear trend at point (LTAP) examination. Missing values were estimated by SPSS, based on the whole study population. The baseline values of all parameters were compared between the study groups, using the unpaired Student’s *T*-test. Testing for changes of the primary study objectives on bone mineral density between the study groups was performed by using an Analysis of the Variances, with the baseline values as Covariate (ANCOVA). Differences between the examination at the baseline level and after the 12-month intervention within the study groups were carried out by the paired Student’s *T*-test. Alterations in the bone blood biomarkers, P1NP and CTX 1, within the study groups, were tested using the Student’s *T*-test for paired samples. All the tests in the descriptive analysis were performed as two-sided tests; the levels of significance were assessed to α = 0.05 at any one time. A *p*-value of < 0.05 was considered to indicate statistical significance. As no hierarchy for the two primary end-points had been defined in the protocol, an analysis, according to Bonferroni–Holm, was performed.

## 3. Results

### 3.1. Subjects

A total of 131 women (age 64.3 ± 7.2 years; body mass index, BMI 23.6 ± 3.6 kg/m^2^; BMD spine T-score: −2.4 ± 0.6; BMD femoral neck T-score: −1.4 ± 0.5) were enrolled in the study and were included in the statistical analysis (ITT). Twenty-nine women dropped out during the study; there were no significant differences in the number of drop-outs between the groups. None of the drop-outs were related to any side effects caused by the intake of the specific collagen peptides or the placebo. No adverse events were noted, and, in particular, no pathological findings were observed due to the intake of the test substances.

To evaluate the homogeneity of the data, at baseline, between the SCP and placebo group, all parameters were compared between both study groups. At the baseline visit, the participants in the SCP group had statistically significant lower bone densities in the spine, compared to the placebo group (*p* = 0.005; Table 1). Therefore, an ANCOVA model was chosen for the statistical analysis, thereby considering the imbalance in baseline data. Also, BMI, despite being in the normal range in both groups, was significantly different at baseline. For all the other data, no statistically significant differences between the study groups were determined at baseline (Table 1).

### 3.2. Changes in Bone Mineral Density

During the course of the study, the bone mineral density increased significantly in the spine (*p* = 0.021) and in the femoral neck (*p* = 0.002) after the SCP treatment (Table 2). In contrast, no significant changes for these parameters were determined in the placebo group (*p* = 0.185 and *p* = 0.552 respectively; Table 2).

**Figure 3:**
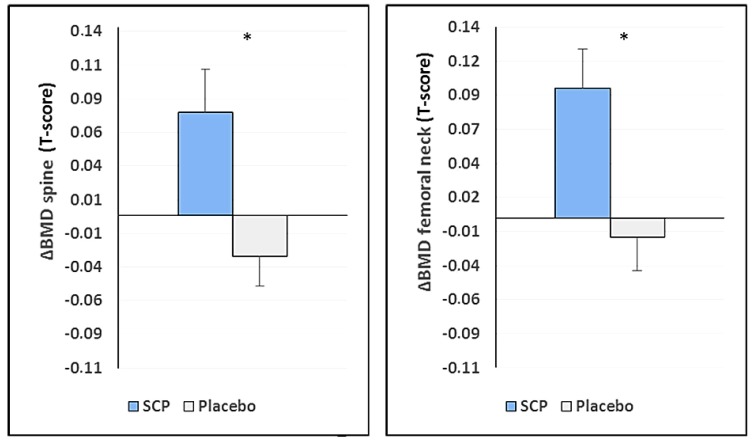
Changes in bone mineral density at the end of the study (ΔBMD X_12_-X_0_). The data represent mean ± SEM, * ANCOVA statistically significantly different. SCP = specific collagen peptides.

The differences observed between the SCP group and the placebo group were verified by analysis of covariance (ANCOVA), considering the unbalanced baseline values. The analysis showed that bone density significantly (*p* = 0.030) increased in the spine and the femoral neck after SCP treatment compared to placebo (*p* = 0.003; Table 3). In the SCP group, BMD increased by almost 3.0% in the spine and 6.7% in the femoral neck, whereas, in the same period, bone density decreased in the placebo group (−1.3% for spine and −1.0% in the femoral neck) (Figure 3).

As no priority or hierarchy had been defined in the study protocol for the two primary end-points, analysis according to Bonferroni–Holm was carried out. The calculated *p*-values in Table 2 were listed in an ascending ranking order, and the statistical significance was evaluated by a Bonferroni–Holm correction. It was demonstrated that treatment with SCP led to a statistically significant increase in the bone density of both primary study end-points “BMD changes in the spine” and “BMD changes in the femoral neck”, as assessed by DXA scans (Figure 2). As the calculated *p*-values for both parameters of the predefined primary end-points are smaller than the *p*-values calculated according to the rules of Bonferroni–Holm, it can be concluded that there was a statistically significant difference between SCP treatment and that of the placebo group, with SCP being superior to placebo. This result was confirmed by the pronounced effect size of the SCP supplementation (d = 0.473 for spine and d = 0.504 for the femoral neck) compared to placebo treatment. 

### 3.3. Bone Biomarkers

Blood samples were analyzed to evaluate specific biomarkers for bone formation and degradation in both treatment groups. At baseline, bone specific amino-terminal propeptide of type I collagen (P1NP) and C-telopeptide of type I collagen (CTX 1) were similar between the study groups.

During the course of the study, P1NP significantly increased in the SCP group (*p* = 0.007), indicating a stimulation of bone formation. In contrast, in the placebo group, no changes in P1NP concentration were determined (*p* = 0.248; Table 3), whereas the bone degradation marker, CTX 1, significantly increased (*p* = 0.011). In contrast, in the participants that were treated with SCP, no changes in bone degradation markers could be determined (*p* = 0.747).

### 3.4. Dietary Intake and Physical Activity

There were no significant baseline differences between the two groups, with respect to macronutrients, calcium and vitamin D (Table 4). However, vitamin D intake was lower than recommended (20 µg/day) in both study groups, before as well as after, the intervention (intake of calcium and vitamin D supplements were not considered in the analysis). Furthermore, absolute protein and fat intakes were significantly above the reference values for nutrient intake. Physical activity and caloric expenditure tended to increase throughout the 12-month study period, but changes did not reach significance (data not shown).

### 3.5. Blood and Safety Parameters

Blood safety parameters (i.e., hemogram, kidney, liver and inflammatory parameters) showed no clinically relevant changes during the course of the 12-month treatment. However, systolic blood pressure (134.5 ± 16.8 vs. 128.1 ± 12.9 mmHg; *p* < 0.001) and also diastolic blood pressure (81.2 ± 9.6 vs. 78.6 ± 7.0 mmHg; *p* = 0.015) were significantly lower in the SCP group after the intervention, as calculated by paired Student’s *t*-tests. In the placebo group, blood pressure remained rather constant.

## 4. Discussion

The main outcome of this randomized, double-blinded and placebo-controlled study in postmenopausal women was that specific collagen peptides significantly increased bone mineral density (BMD) in both the lumbar spine and femoral neck. In contrast, no significant changes for these parameters were determined in the placebo group. Considering the decrease in BMD in the control group, subjects in the SCP group showed a 4.2% higher BMD in the spine and a 7.7% higher BMD in the femoral neck, suggesting a clinically relevant effect of the 12-month treatment with SCP [22]. The anabolic effect of SCP intake was also confirmed by a significant increase in the bone formation biomarker, amino-terminal propeptide of type I collagen (P1NP). In contrast, only in the control group, a significant increase in the concentration of the bone degradation marker C-telopeptide of type I collagen (CTX 1) could be detected after the 12-month study period.

To the author’s knowledge, only two studies have investigated the effect of collagen peptides on bone markers and bone mineral density in humans. In one study, the effects of collagen peptides were investigated, with and without calcitonin [14]. The authors measured urinary pyridinoline cross-links and suggested, from their results, that calcitonin plus collagen peptides had a greater effect on the inhibition of bone collagen breakdown. Another investigation, using a supplement with a combination of collagen + calcium + vitamin D, found that the loss in BMD was substantially lower in the collagen supplemented group than in the group with calcium + vitamin D alone [23].

Direct scientific evidence to explain the positive effects of collagen supplementation in humans is still lacking. However, some findings from cell experiments and in vivo studies in rodents have enhanced our knowledge of how collagen peptides could enhance bone formation and increase BMD. First of all, it has been shown that collagen peptides are rapidly absorbed from the gastrointestinal tract [24,25]. In addition, collagen peptides are absorbed in the small intestine to a considerable amount in peptide form and may act as signaling molecules, thereby positively influencing anabolic processes [25,26]. Especially for connective tissue, this stimulating effect has previously been demonstrated [27,28,29]. With respect to myoblast differentiation and myotube hypertrophy, Kitakaze et al. identified the dipeptide (Hyp-Gly) from collagen as a signaling peptide that activates the PI3K/Akt/mTOR pathway [30]. Therefore, it may be assumed that also the stimulation of collagen formation in the bone could be mediated via signaling proteins derived from collagen peptides. Collagen peptides have been shown to increase gene expression of collagen type 1, alpha 1 (COLIA1). In addition, the authors found that the ERK/MAPK signaling pathway was involved in the collagen-induced increase in COLIA1 expression [31]. Collagen is by far the major constituent of bone mass. Results from rodent studies have demonstrated that collagen peptides significantly increase the organic substance of the bone [9]. Therefore, an increase in this organic fraction and the following mineralization of the bone may result in an increased BMD. In the present study, an anabolic effect, with respect to collagen synthesis and bone anabolism, was reflected by an increase in the bone marker P1NP in the SCP supplemented group. In contrast, the bone degradation marker, CTX1, was significantly increased in the control group only. Comparable results were also found in ovariectomized rats, following oral administration of bovine collagen peptides [32].

Other animal studies have shown that collagen peptides or gelatin hydrolysates increase the longitudinal bone growth in rats [33], increase the bone mass in both growing rats following treadmill training [34] as well as mature rats [35], inhibit bone loss in ovariectomized rats [12,36] and prevent bone loss in estrogen-deficient rats, probably by reducing the levels of proinflammatory cytokines [11].

Together, the increasing knowledge about the signaling characteristics of collagen peptides [30,31,37], as well as the aforementioned encouraging findings in animal studies, make the results of the current investigations comprehensible, although considerably more data are needed from human studies.

Nevertheless, there is still insufficient knowledge about which type of collagen peptides (marine, porcine, bovine etc.) exerts the most favorable effect. Moreover, not all collagen peptides may have the same effects in different kind of diseases, and, finally, the manufacturing process could also have an influence on the biological and physiological properties of collagen peptides and thus, their effectiveness.

Apart from the fact that we need more and larger human studies, we also need additional data regarding the optimal timing and dosage as well as findings related to longer-term effects of supplementation with collagen peptides.

## 5. Conclusions

In conclusion, the findings of this randomized, placebo-controlled trial demonstrate, that supplementation with 5 g of specific collagen peptides significantly increases bone mineral density of the lumbar spine and the femoral neck as well as blood levels of the bone marker, P1NP, in postmenopausal women with age-related decline in BMD.

## Figures and Tables

**Table 1:** Baseline data [t_0_].

	SCP (*n* = 66)	Placebo (*n* = 65)	*p*-Value
Age [years]	63.8 ± 7.4	64.9 ± 7.1	0.378
Height [m]	1.64 ± 6.8	1.62 ± 7.0	0.222
BMI	24.4 ± 3.7	22.9 ± 3.4	0.016
BMD spine [T]	−2.5 ± 0.6	−2.3 ± 0.6	0.005
BMD femoral neck [T]	−1.4 ± 0.5	−1.4 ± 0.5	0.903

The data represent mean ± SD. BMI, body mass index; SCP, specific collagen peptides.

**Table 2:** Overview of the T-scores at t_0_, t_12_ for each treatment group.

	Group	*n*	${\bar{X}}_{0}$ ± SD_0_	${\bar{X}}_{12}$ ± SD_12_	*p*-Value *	ANCOVA *p*-Value
BMD spine [T]	SCP	66	−2.54 ± 0.6	−2.47 ± 0.6	0.021	0.030
	Placebo	65	−2.25 ± 0.6	−2.28 ± 0.6	0.185	
BMD femoral neck [T]	SCP	66	−1.41 ± 0.5	−1.32 ± 0.5	0.002	0.003
	Placebo	65	−1.42 ± 0.5	−1.44 ± 0.5	0.552	

The data represent mean ± SD, * Paired Student’s test. ${\bar{X}}_{0}$ = initial examination (t_0_); ${\bar{X}}_{12}$ = examination after 12 months (t_12_).

**Table 3:** Changes in bone biomarkers, between t_0_ and t_12_, in the confirmatory study groups.

	Group	*n*	${\bar{X}}_{0}$ ± SD_0_	${\bar{X}}_{12}$ ± SD_12_	*p*-Value *
P1NP [ng/mL]	SCP	66	33.34 ± 24.70	37.22 ± 27.70	0.007
	Placebo	65	38.74 ± 27.00	40.6 ± 28.35	0.248
CTX 1 [ng/mL]	SCP	66	0.81 ± 0.40	0.80 ± 0.35	0.747
	Placebo	65	0.68 ± 0.31	0.80 ± 0.58	0.011

* Paired Student’s *T*-test. CTX 1, C-telopeptide of type I collagen; P1NP, amino-terminal propeptide of type I collagen.

**Table 4:** Intake of energy and nutrients compared to recommended dietary allowance (RDA) (US) [20,21].

	Group	${\bar{X}}_{0}$ ± SD_0_	${\bar{X}}_{12}$ ± SD_12_	RDA US (Women 51–70 Years)
Energy [kcal]	SCP	1978 ± 387	2010 ± 485	2403 (19 years) ^1^
	Placebo	2029 ± 524	2030 ± 576	
Protein [g]	SCP	75 ± 18 ^a^	73 ± 19 ^a^	46 (≙0.8 g/kg BW)
	Placebo	85 ± 39 ^a^	87 ± 29 ^a^	
Fat [%]	SCP	35 ± 7	34 ± 7	ND
	Placebo	38 ± 7	34 ± 7	
Calcium [mg]	SCP	1086 ± 262	1064 ± 348	1200
	Placebo	1383 ± 590	1220 ± 466	
Vitamin D [µg]	SCP	1.94 ± 2.9 ^b^	2.13 ± 2.4 ^b^	15
	Placebo	2.92 ± 2.8 ^b^	4.00 ± 3.1 ^b^	

^a^ = Significant higher intake than recommended, ^b^ = significant lower intake than recommended, ND = not determined, ^1^ = For healthy active Americans and Canadians; subtract 7 kcal/day for females for each year of age above 19 years, BW, bodyweight.
